# Supplementary figures and images for: MCU-enriched dendritic mitochondria regulate plasticity in distinct hippocampal circuits
Source: bioRxiv. 2024 Apr 3:2023.11.10.566606. Originally published 2023 Nov 11. Preprint. [Version 2] doi: 10.1101/2023.11.10.566606 (PMC10659405; doi:10.1101/2023.11.10.566606)

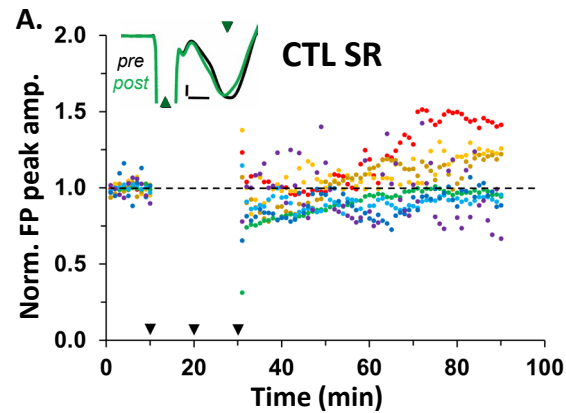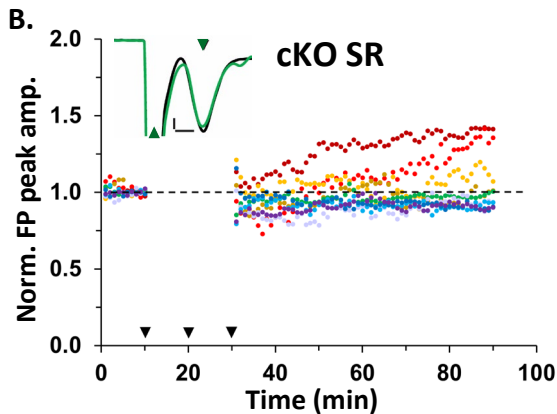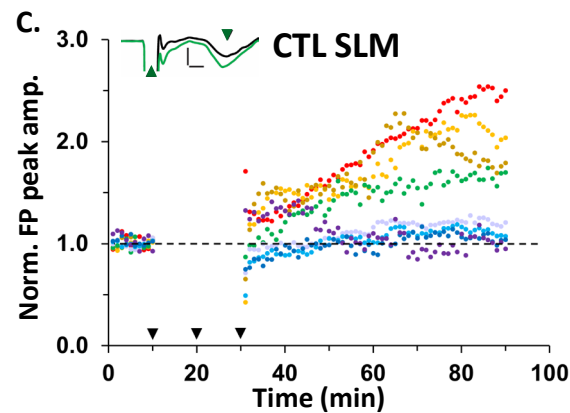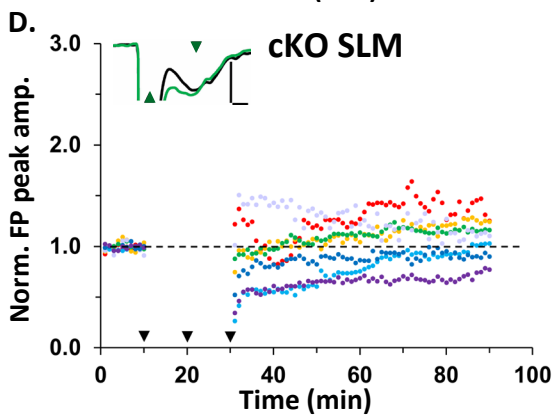

Supplement: Supplement 1 — A. Individual time plots of normalized FP peak amplitudes evoked by stimulation of SC inputs to SR of CTL slices. Individual plots are color-coded according to post/pre ratio, with progressively warmer colors representing progressively higher post/pre ratios (all panels). Black dotted line = baseline (all panels). Black arrowheads = stimulation at 100 Hz for 1s; 3 bursts with 10 min interval. Inset shows a representative recording of the average evoked response recorded during the last 5 minutes of pre-conditioning (black line) versus the last 5 minutes of post-conditioning (green line; all panels). Green up arrowhead = stimulus artifact; green down arrowhead = FP. Scale bars: 0.1mV, 0.5ms (all panels). B. Individual time plots of normalized FP peak amplitude evoked by stimulation of SC inputs to SR of cKO slices. C. Individual time plots of normalized FP peak amplitude evoked by stimulation of PP inputs to SLM of CTL slices. D. Individual time plots of normalized FP peak amplitude evoked by stimulation of PP inputs to SLM of cKO slices. [file media-1.pdf]

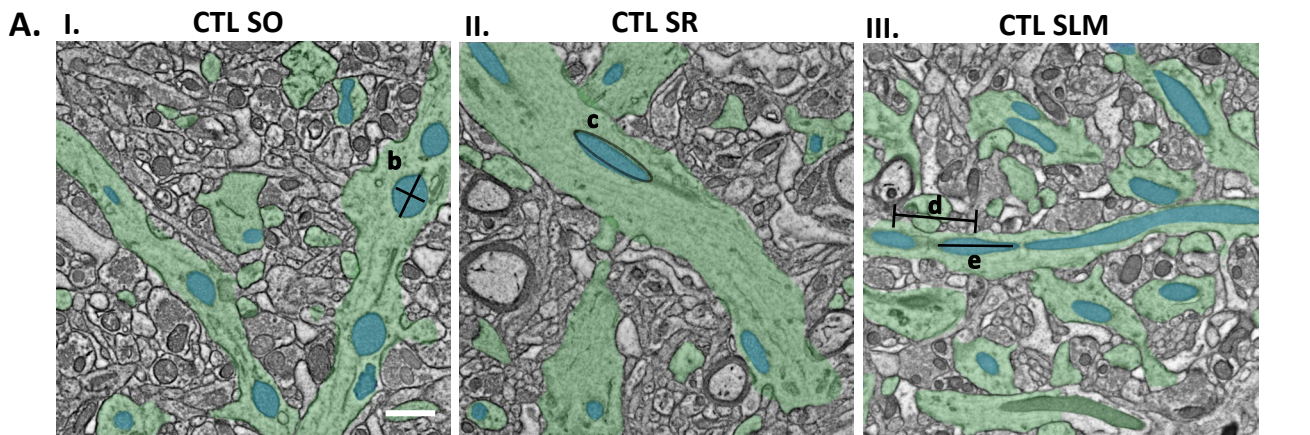

**b. Aspect Ratio** **c. Area** **d. Nearest Neighbor Distance** **e. Feret's Diameter**

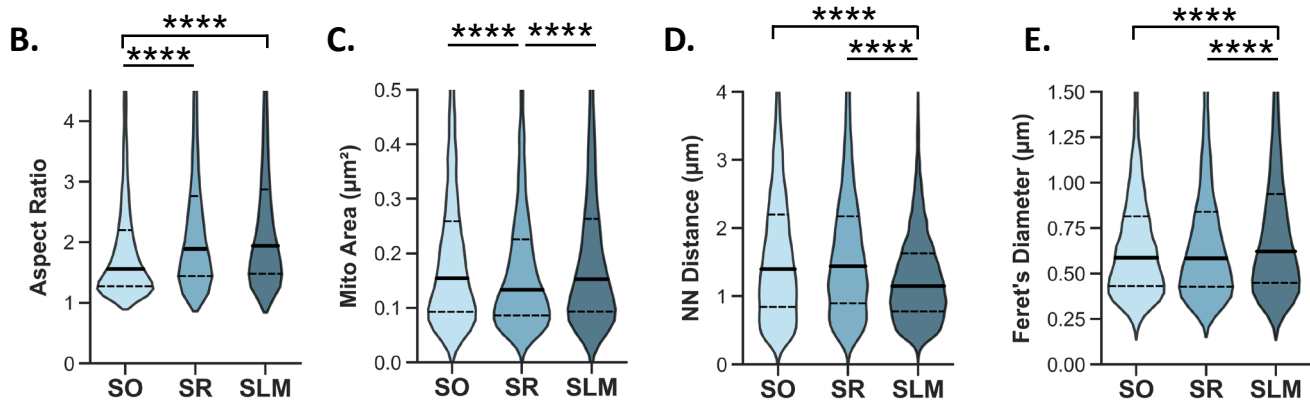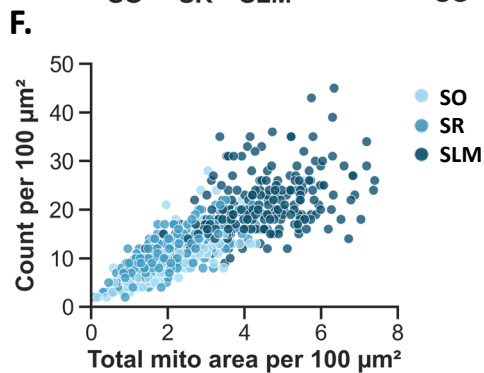

Supplement: Supplement 2 — A. Representative SEM images showing dendritic mitochondria (blue) and dendrites (green) in CA2 SO, SR and SLM of CTL mice. Examples of the measured metrics are illustrated. Scale bar = 1 μm. B. Mitochondrial aspect ratio in CA2 SO, SR, and SLM of CTL. Overall effect of layer was significant (two-way ANOVA, F (2, 23653) = 203.2, p < 0.0001; SO n = 2353, SR n = 2904, SLM n = 5236 mitochondria, 3 mice). C. Individual mitochondria area in the same dataset as in (B). Overall effect of layer was significant (two-way ANOVA, F (2, 23653) = 131.7, p < 0.0001). D. Mitochondria nearest neighbor distance in the same dataset as in (B). Overall effect of layer was significant (Two-way ANOVA, F (2, 23653) = 570.00, p < 0.0001). E. Mitochondria Feret’s diameter in the same dataset as in (B). Overall effect of layer was significant (two-way ANOVA, F (2, 23653) = 162.6, p < 0.0001). F. A correlation of mitochondrial count and total mitochondrial area per 100 μm2 image tile in CA2 SO, SR, and SLM. Overall effect of layer was significant for both mitochondria count (two-way ANOVA; F (2, 1551) = 590.35, p < 0.0001) and total mitochondria area (two-way ANOVA; F (2, 1551) = 976.34, p < 0.0001; SO n = 223, SR n = 279, SLM n = 260, n = 100 μm2 tiles). Mann Whitney post hoc tests and Sidak’s correction comparing layers are shown. For all violin plots, solid line = median; dashed line = upper and lower quartiles. [file media-2.pdf]

Area

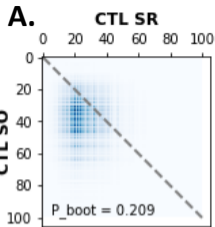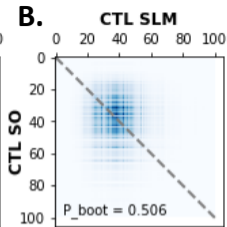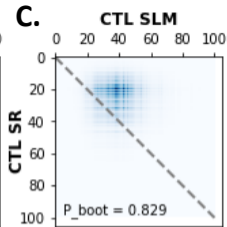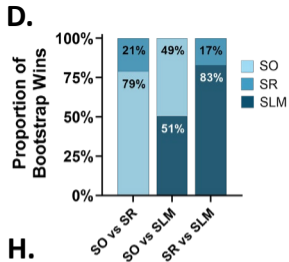

Count

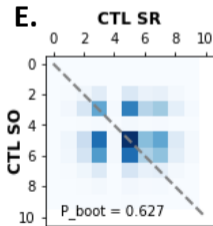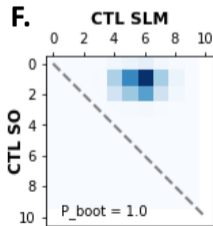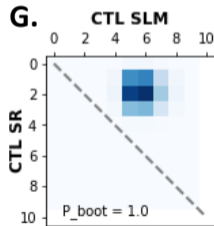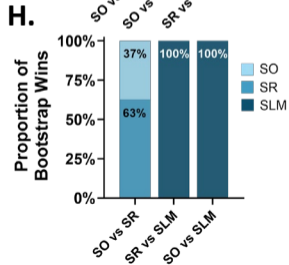

Supplement: Supplement 3 — A-C. Comparison of resampled mitochondria area medians across SO and SR (A), SO and SLM (B) or SR and SLM (C) of CTL CA2 with a probability distribution plot. P_boot = the proportion of medians within the upper triangle of the plot. N = 10,000 repetitions. Data was binned into 100 equal bins. D. Bar plot showing the proportion of bootstrap repetitions where mitochondria area was greater in SO, SR or SLM for each comparison. E-G. Comparison of resampled median mitochondria count per tile across SO and SR (E), SO and SLM (F) or SR and SLM (G) of CTL CA2. N = 10,000 repetitions. Data was binned into 10 equal bins. H. Bar plot showing the proportion of bootstrap repetitions where mitochondria count per tile was greater in SO, SR or SLM for each layer comparison in CTL CA2. [file media-3.pdf]

Area

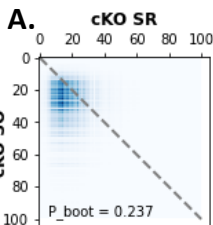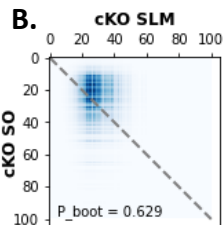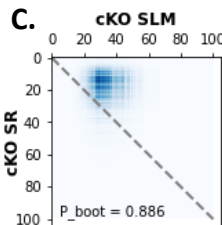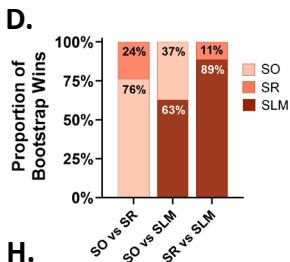

Count

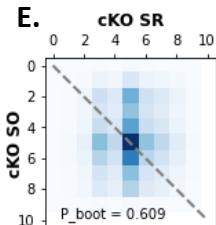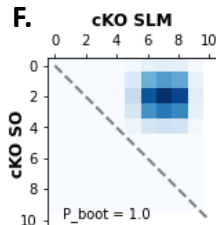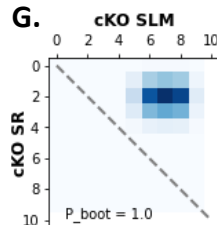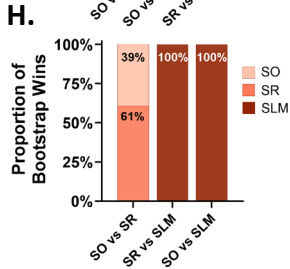

Area

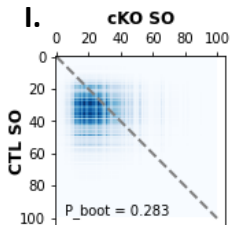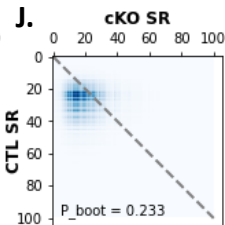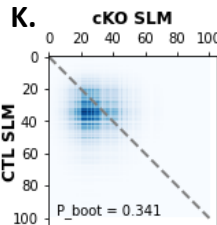

Count

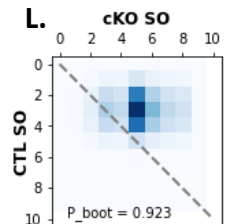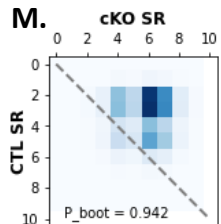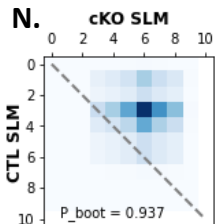

Supplement: Supplement 4 — A-C. Comparison of resampled mitochondria area medians across SO and SR (A), SO and SLM (B) or SR and SLM (C) of CA2 in MCU cKO mice. P_boot = the proportion of medians within the upper triangle of the plot. N = 10,000 repetitions. Data was binned into 100 equal bins. D. Bar plot showing the proportion of bootstrap repetitions where mitochondria area was greater in SO, SR or SLM for each layer comparison in the cKO. E-G. Comparison of resampled median mitochondria count per tile across SO and SR (E), SO and SR (F) or SR and SLM (G) in CA2 of MCU cKO mice. N = 10,000 repetitions. Data was binned into 10 equal bins. H. Bar plot showing the proportion of bootstrap repetitions where mitochondria count per tile was greater in SO, SR or SLM for each layer comparison. I-K. Comparison of resampled mitochondria area medians in the cKO and CTL for SO (I), SR (J) and SLM (K). This is the same bootstrap population as in Figure 4I. L-N. Comparison of resampled mitochondria count medians in the cKO and CTL for SO (L), SR (M) and SLM (N). This is the same bootstrap population as in Figure 4J. [file media-4.pdf]
