# Supplementary material for: The mitochondrial calcium uniporter is necessary for synaptic plasticity and proper mitochondrial morphology and distribution in the distal dendrites of CA2 neurons
Source: bioRxiv. 2023 Nov 11:2023.11.10.566606. Preprint. [Version 1] doi: 10.1101/2023.11.10.566606 (PMC10659405; doi:10.1101/2023.11.10.566606)
Supplement: Supplementary file 1 [file media-1.pdf]

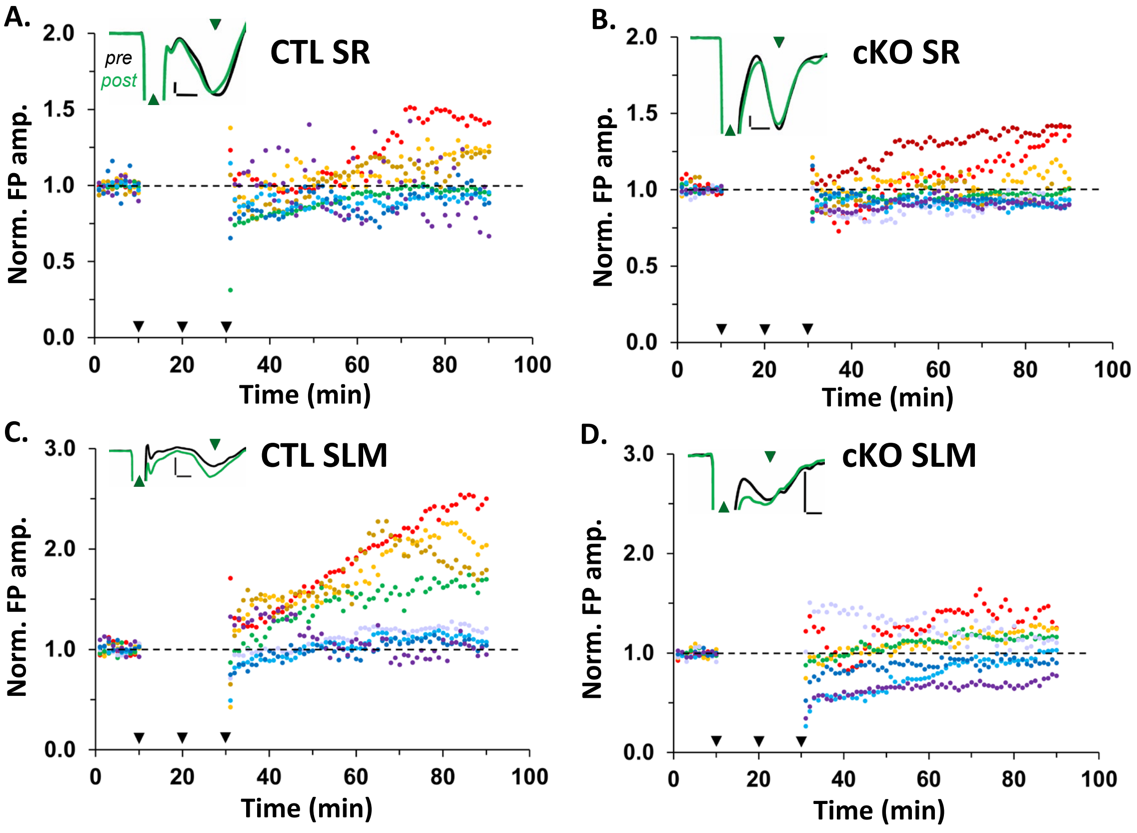

A.
CTL SR
pre
post
Norm. FP amp.
Time (min)
B.
cKO SR
Norm. FP amp.
Time (min)
C.
CTL SLM
Norm. FP amp.
Time (min)
D.
cKO SLM
Norm. FP amp.
Time (min)
