# Supplementary material for: The mitochondrial calcium uniporter is necessary for synaptic plasticity and proper mitochondrial morphology and distribution in the distal dendrites of CA2 neurons
Source: bioRxiv. 2023 Nov 11:2023.11.10.566606. Preprint. [Version 1] doi: 10.1101/2023.11.10.566606 (PMC10659405; doi:10.1101/2023.11.10.566606)
Supplement: Supplementary file 2 [file media-2.pdf]

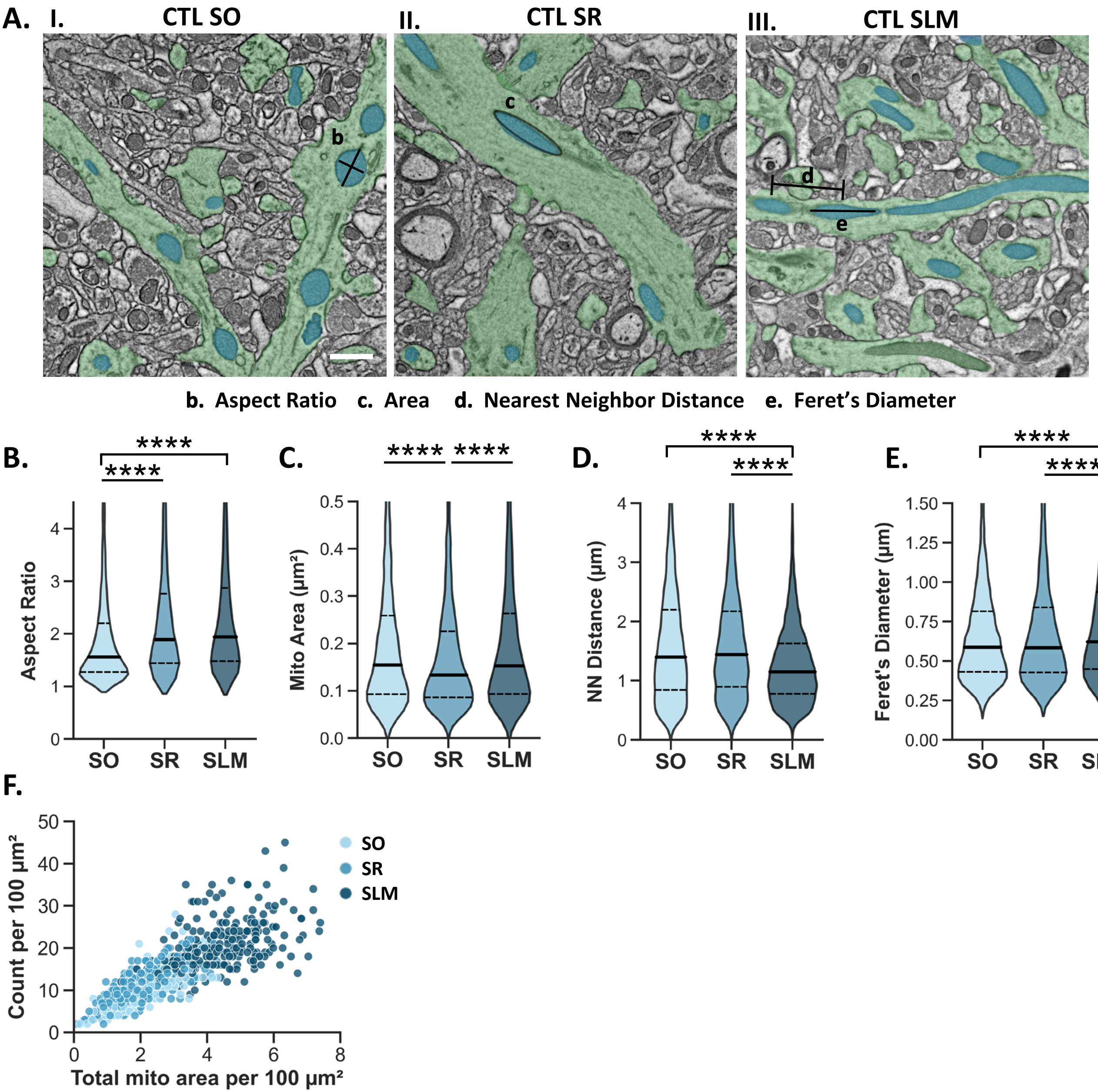
A.
I. CTL SO
II. CTL SR
III. CTL SLM
b
c
d
e
b. Aspect Ratio c. Area d. Nearest Neighbor Distance e. Feret's Diameter
B.
C.
D.
E.
****
Aspect Ratio
Mito Area (µm²)
NN Distance (µm)
Feret's Diameter (µm)
SO
SR
SLM
F.
Count per 100 µm²
Total mito area per 100 µm²
SO
SR
SLM
